# Psychological Predictors of Energy Saving Behavior: A Meta-Analytic Approach

**DOI:** 10.3389/fpsyg.2021.648221

**Published:** 2021-06-24

**Authors:** Giuseppe Carrus, Lorenza Tiberio, Stefano Mastandrea, Parissa Chokrai, Immo Fritsche, Christian A. Klöckner, Torsten Masson, Stepan Vesely, Angelo Panno

**Affiliations:** ^1^Department of Education, Experimental Psychology Laboratory, Roma Tre University, Rome, Italy; ^2^Department of Social Psychology, Institute of Psychology, University of Leipzig, Leipzig, Germany; ^3^Department of Psychology, Faculty of Social and Educational Sciences, Norwegian University of Science and Technology, Trondheim, Norway; ^4^Department of Human Science, European University of Rome, Rome, Italy

**Keywords:** meta-analysis, energy saving behaviors, attitudes, intentions, values, awareness, emotions

## Abstract

Understanding how psychological processes drive human energy choices is an urgent, and yet relatively under-investigated, need for contemporary society. A knowledge gap still persists on the links between psychological factors identified in earlier studies and people's behaviors in the energy domain. This research applies a meta-analytical procedure to assess the strength of the associations between five different classes of individual variables (i.e.,: attitudes, intentions, values, awareness, and emotions) and energy-saving behavioral intentions and behaviors (self-reported and actual). Based on a systematic review of studies published between 2007 and 2017, we estimate the average effect size of predictor-criterion relations, and we assess relevant moderators and publication bias, drawing on data obtained from 102 independent samples reported in 67 published studies (*N* = 59.948). Results from a series of five single meta-analyses reveal a pattern of significant positive associations between the selected psychological determinants and energy-saving indicators: associations between individual-level predictors and energy-saving outcomes are positive and moderate in size, ranging from large effects for emotions to small-moderate effects for pro-environmental values. Interestingly, moderation analysis reveals, among other things, that attitude-behavior links are not statistically significant when actual behavior is considered as an outcome. Implications for policy interventions are discussed.

## Introduction

Climate change is currently a central part of the global energy debate and public discourse. Climate scientists agree that climate change is caused by the considerable increase in the concentration of greenhouse gases in the atmosphere, directly or indirectly attributable to humans' use of fossil fuels. It is therefore necessary to underline the need to change our energy consumption behaviors not only individually, but also collectively. From the point of view of environmental psychological science, addressing climate change is considered as a fundamental challenge, which requires a deep understanding of the psychological processes involved in both pro-environmental behaviors and lifestyles in general, and human energy consumption in particular (e.g., Clayton, 2020; Hartig, 2020; Bouman and Steg, 2020).

The purpose of this paper is to present an overarching view of published empirical research on the relation between psychological factors and energy-related choices and behaviors. We argue here that a meta-analytical study of this kind could be useful for both scientists and decision makers in the energy domain and contribute to build on the currently available knowledge on the human dimension of the sustainable energy transition (e.g., Steg et al., 2015; Tiberio et al., 2020). Some interesting systematic reviews and meta-analyses on these issues have recently appeared, highlighting for example the role of identity variables on a wide range of pro-environmental behaviors, which include, but are not limited to, energy-related ones (e.g., Fritsche et al., 2018). Other works have attempted to focus more specifically on energy-saving behaviors, mostly through systematic literature reviews, in order to identify the general factors that might influence them (including psychological determinants: see for example Steg, 2008; Yang et al., 2016). Other contributions assessed the effects of behavioral intervention strategies (e.g., Abrahamse et al., 2005; Abrahamse and Steg, 2013; Delmas et al., 2013; Karlin et al., 2015; Andor and Fels, 2018; Bergquist et al., 2019; Nisa et al., 2019; Buckley, 2020). However, apart from some notable comprehensive overviews (e.g., Steg et al., 2015) or broader contributions (van Valkengoed and Steg, 2019), to our knowledge, there are no recent meta-analyses or systematic reviews that have focused on the direct psychological predictors of energy-related behaviors. Therefore, drawing on literature from a broad spectrum of studies across psychological sciences, in this paper we identify five categories of psychological variables that have been acknowledged in previous studies as key factors for explaining variability in energy-saving behavior. Individual level factors such as ecological attitudes, pro-environmental values, awareness of consequences of one's behavior and beliefs in climate change, emotions, and intentions to adopt energy-saving solutions have been frequently considered as potential antecedences of energy-saving behaviors. In this paper, we use a meta-analytical procedure to assess the strength of the associations between five different classes of individual psychological variables (i.e.,: attitudes, values, intentions, awareness, and emotions) and energy-saving behavioral behaviors (self-reported and actual). To conduct such a meta-analysis, the goal of our literature search was to identify published empirical studies that examined the links between attitudes, intentions, values, awareness and emotions on the one hand, and people's observed and actual behaviors in the energy domain, on the other hand. In this work, the intention to adopt energy-saving solutions has been considered both as predictor of self-reported and actual energy-saving behavior, or as an outcome, when either self-reported or actual behavior were not available in the primary studies considered. Indeed, a large number of studies use intentions as their only outcome of antecedent factors aimed to explain the adoption of energy-saving solutions, assuming that it can be considered as a reliable proxy of behavior in the energy domain.

In the next sections we briefly review the literature behind each of these classes of predictors, and we present and discuss the results of the meta-analytical tests conducted.

## Theoretical Background

### Attitudes

The main reason for studying environmental attitudes in the field of energy saving behavior is related to the well-known attitude- behavior link in social psychology. Positive attitudes toward a specific environmental issue (e.g., climate change) were found to be associated to behavioral intention in that same domain (e.g., Poortinga et al., 2004). Widely used theories and models, such as the Theory of Planned Behavior (TPB; Ajzen, 1991) have explained the attitude-behavior link, and the circumstances under which it occurs, both in general (Manstead, 1996) and in the environmental domain in particular (Staats, 2003), In the specific energy-related domain, the TPB framework has been applied to analyse both individual's energy saving behaviors as well as the acceptance of renewable energy technologies (Abrahamse and Steg, 2011; Wang et al., 2011; Alam and Rashid, 2012). Studies in this field report a positive association between attitudes toward electric cars and different adoption indicators (Moons and De Pelsmacker, 2012; Nayum and Klöckner, 2014; Barbarossa et al., 2015; Degirmenci and Breitner, 2017).

While numerous studies suggest a strong association between attitudes and behavior in the environmental domain, other authors highlight the poor predictability of behavior from attitudes; this inconsistency is usually referred to as the attitude-behavior gap (Gifford and Sussman, 2012). A possible explanation of this discrepancy lies in the choice of the methods of collecting behavioral data. The most common method in social research is self-reported behavior, through questionnaires and other measures that frequently do not reflect the actual adoption of a behavior and are more subject to a social desirability bias (Gifford and Sussman, 2012). This aspect suggests the plausibility of moderating factors intervening in the relation between attitudes and behaviors in the energy domain such as the actual vs. self-report measurement method.

### Intentions

Behavioral intention is commonly assumed to be an immediate antecedent of behavior (Ajzen, 1991), although that does not mean that intentions always predict behaviors (e.g., Sheeran, 2002; Webb and Sheeran, 2006; Frederiks et al., 2015). Intention serves as a presupposition of favorable energy-saving choices and encompasses the likelihood of a specific course of action, such as for example purchasing a particular energy-efficient product or adopting specific energy-saving solutions as a result of environmental needs.

Energy-related intentions were in fact seen to have a moderate positive association with energy efficiency behaviors (e.g., Zierler et al., 2017). Afroz et al. (2015a) found a link between intention and behavior in the purchase of environmentally friendly vehicles. A moderate, although indirect, effect of behavioral intention was found also on purchase decisions in relation to LED technology adoption, in a study by Khorasanizadeh et al. (2016). Thus, it is worth to include intentions in our meta-analysis as a factor to be estimated as a potentially relevant predictor of energy-related choices.

### Values

The role of human values in pro-environmental behaviors has been often deemed as fundamental. Some values can hinder pro-environmental actions, other values can encourage the adoption of more sustainable ones (Steg and De Groot, 2012). A widely cited model in the literature, such as the value-belief-norm theory (see Stern et al., 1999) emphasizes the indirect association between values and decisions about the environment. Many studies showed associations between biospheric value orientations and specific energy related behaviors such as, for example, residential energy usage (Schultz, 2000; Abrahamse and Steg, 2009, 2011). Thus, in our meta-analysis, it is worth considering the link between biospheric values and energy-saving behavior.

Other studies also showed that altruistic or self-transcendent values (as opposed to self-enhancement ones) are linked to pro-environmental attitudes and behaviors (Nordlund and Garvill, 2002; Schultz et al., 2005; Collins et al., 2007). In particular, the study by Schultz et al. (2005) was conducted across six different countries, involving around 720 participants, and showed that self-transcendent values are positively related to environmental concern, while self-enhancement values are negatively related to general concern, consistently across different cultures.

In sum, values have been commonly related to human behavior in the energy domain. However, as in the case of attitudes and knowledge, a “value-action gap” should also be taken into account (e.g., Huddart-Kennedy et al., 2009). Daily life presents many situations where people endorsing values promoting the mitigation of negative consequences of environmental problems (e.g., global warming, climate change) and the adoption of energy-saving solutions or “low carbon” technologies (such as renewable energy sources) fail to translate these values, beliefs and attitudes into practical actions in their daily life choices. It is therefore important to systematically assess the strength of the relation between value endorsements and energy-related behavior.

### Awareness

For the purposes of this paper, under the label “awareness” we group together aspects that have been linked to individuals' energy choices, such as knowledge of environmental facts, awareness of the consequences of one's own behavior, or beliefs about climate change or global warming. Although people's direct knowledge about environmental issues is usually limited, it has been argued that “high level of awareness enables individuals to make conscious choices for acting in an environmentally friendly way” (e.g., Partanen-Hertell et al., 1999, p. 9). Environmental awareness has also been defined in terms of environmental knowledge and/or recognition of environmental problems (Grob, 1995). In our meta-analysis, we refer to those environmental problems that derive from the effects of global climate change and to public's awareness of adverse consequences of environmental problems. The awareness of consequences (or increasing knowledge) is also an important factor identified in widely-studied models of pro-environmental action, such as the Value–Belief–Norm theory (Stern et al., 1999) or Norm Activation Model (Schwartz, 1977). Indeed, previous studies documented an increase in the public awareness of adverse consequences of climate change (e.g., Ockwell et al., 2009; Steg, 2008). Although it has been suggested that “while awareness about the issue is now very high, climate change continues to be a low priority issue for most people” (Whitmarsh, 2011, p. 691), it is arguable that being aware of climate change facts or global warming trends can impact individual energy-related decisions. A 2009 survey of the UK Department for Environment, Food and Rural Affairs found for example that the majority of respondents claimed that they were trying to cut down on the use of gas and electricity at home in response to the threats of climate change (see Thornton, 2009). Indeed, awareness of consequences has been shown to increase the intention to adopt an electric vehicle (Bockarjova and Steg, 2014), or to curtail energy consumption (van der Werff and Steg, 2015). Likewise, people with higher awareness of consequences have been identified as more likely to adopt an electric car (Nayum et al., 2016). In their meta-analysis, Bamberg and Möser (2007) suggest, however, that awareness is an important but indirect determinant of pro-environmental behavioral intentions: this seems to be somehow corroborated by recent contradictory and partly surprising findings (e.g., Whitmarsh et al., 2020).

### Emotions

Emotions have a crucial role in motivating human behavior (Damasio, 1994; LeDoux, 2012; Levine and Leven, 2014), including pro-environmental and energy-related behaviors (Hine et al., 2007; Carrus et al., 2008; Ferguson and Branscombe, 2010; Onwezen et al., 2013; Rees et al., 2015).

For example, anticipated emotions can be a direct cause of human behavior: an individual's ability to appraise a future emotional state enables to elaborate and to assess the value of the potential outcomes of one's own behavior (e.g., Panno et al., 2015). In fact, it has been shown that people's negative emotions (e.g., anger, frustration, sadness) about engaging in pro-environmental behavior (for example in the area of transport modes choice or waste recycling) reduced their desire to engage in these pro environmental behaviors (Carrus et al., 2008), while positive emotions regarding cycling (e.g., feeling happy and satisfied) increases the desire to choose cycling as transportation mode (Passafaro et al., 2014).

An association between emotions and behavior in the environmental domain was also highlighted for two specific types of discrete emotions: feelings of guilt and pride (e.g., Kaiser, 2006; Elgaaied, 2012). A positive effect of a guilt induction (compared with no emotional induction) emerged in a study on support for climate change policy (Lu and Schuldt, 2015). In an experimental study, Schneider et al. (2017) examined the causal effects of pride vs. guilt on pro-environmental decision making and behavioral intentions, inducing these anticipated emotions just prior to asking participants to make a series of environmental decisions. Results showed that stimulating people to anticipate feelings of pride for positive future pro-environmental actions seems to have a more significant effect compared to prompting feelings of guilt for inactions. Understanding the role of emotions in everyday life energy choices has therefore the potential to help in defining strategies and designing behavioral interventions to promote the sustainable energy transition. However, the study of emotions as antecedents of energy-related behavior received so far a relatively limited attention in the environmental psychological literature. Thus, in this paper, we considered emotions (either anticipated emotions or other types of emotional states) as a relevant predictor of energy-saving.

## Method

### Eligibility Criteria

For the research methodology in this study, we used the Preferred Reporting Items for Systematic Reviews and Meta-Analyses (PRISMA) provided by Moher et al. (2009).

The goal of our literature search was to identify published empirical studies that examined the links between attitudes, intentions, values, awareness and emotions (X) and people's observed and actual behaviors in the energy domain (Y). Thus, in the meta-analysis we included papers that reported firsthand data about the relationship between X and Y. Technically speaking, we conducted five separate meta-analyses between variable pairs. We completed the literature search on June 20th, 2017. Various criteria were applied to select eligible data for inclusion in the analysis. Specifically, studies were included in the meta-analysis if: (1) they were published in a peer-reviewed journal in the last 10 years; (2) they were published in English; (3) the dependent variable was an energy-saving behavior (actual or self-reported) or an energy-saving behavioral intention; (4) among the independent variables there was at least one of the following measures: attitudes, pro-environmental values, awareness, emotions, intentions (intentions were considered as predictors only for studies where the criterion variable was behavior); (5) in case of studies using an experimental design, the studies were included only if the experimental design had a control group; (6) in the case of papers where bivariate correlations between the respective dependent and independent variables and the sample size were not reported, we contacted authors to obtain the data via email; in case of no response after two email reminders, the correlations were estimated starting from other data available in the paper, whenever possible (e.g., regression coefficients). When a direct coefficient-based estimation was not possible, the paper was not included in the analysis.

In addition to excluding studies that did not meet the inclusion criteria cited above, we also excluded those studies that, rather than on energy use and consumption, were focused more on ideological, political or social stances that individuals, groups and communities might have in regard to energy-related issues; in this category, there are for example many studies that investigate people's reactions to nuclear energy policies, or people's aesthetic judgements or attitudes toward wind turbines, power lines, and so forth: these kind of studies were not included in our meta-analysis. Finally, qualitative studies that did not provide sufficient statistical data to allow the calculation of an effect size were not included.

### Search Strategies and Study Selection

We conducted the literature search considering a time frame of 10 years (2007–2017). The main strategy consisted of searching two major electronic databases of scientific literature (ScienceDirect and Scopus) using the following search terms:

“(attitude and energ*) or (attitude and electric*) or (emotion* and energ*) or (emotion* and electric*) or (guilt and energ*) or (guilt and electric*) or (pride and energ*) or (pride and electric*) or (anger and energ*) or (anger and electric*) or (“belief* in climate change” and energ*) or (”belief* in climate change“ and electric*) or (”belief* in global climate change“ and energ*) or (”belief* in global climate change“ and electric*) or (”belief* in global warming” and energ*) or (“belief* in global warming” and electric*) or (“belief* of climate change” and energ*) or (“belief* of climate change” and electric*) or (“belief* of global climate change” and energ*) or (“belief* of global climate change” and electric*) or (“belief* of global warming” and energ*) or (“belief* of global warming” and electric*) or (“belief* about climate change” and energ*) or (“belief* about climate change” and electric*) or (“belief* about global climate change” and energ*) or (“belief* about global climate change” and electric*) or (“belief* about global warming” and energ*) or (“belief* about global warming” and electric*) or (“climate change risk perception*” and energ*) or (“climate change risk perception*” and electric*) or (“perception* of climate change ” and energ*) or (“perception* of climate change ” and electric*) or (“climate change perception*” and energ*) or (“climate change perception*” and electric*) or (“knowledge in climate change” and energ*) or (“knowledge in climate change” and electric*) or (“ knowledge in global climate change” and energ*) or (“ knowledge in global climate change” and electric*) or (“ knowledge in global warming” and energ*) or (“ knowledge in global warming” and electric*) or (“knowledge about climate change” and energ*) or (“knowledge about climate change” and electric*) or (“ knowledge about global climate change” and energ*) or (“ knowledge about global climate change” and electric*) or (“ knowledge about global warming” and energ*) or (“ knowledge about global warming” and electric*) or (awareness and energ*) or (awareness and electric*) or (intention* and energ*) or (intention* and electric*) or (“environment* value*” and energ*) or (“environment* value*” and electric*) or (“value system*” and energ*) or (“value system*” and electric*).”

**Figure 1:**
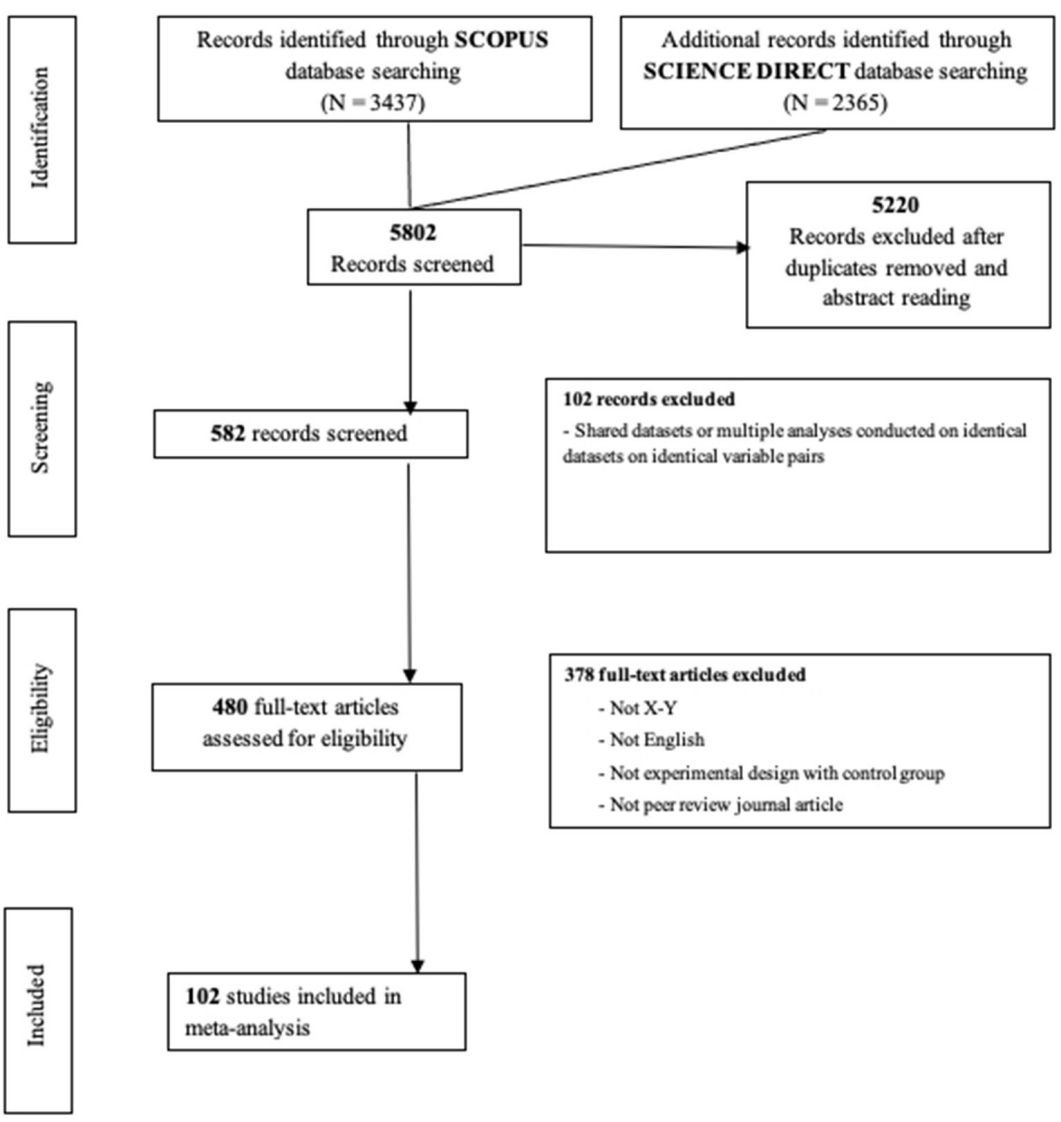
PRISMA diagram describing the article selection phases.

Furthermore, we hand-searched in the references of the selected journal articles further relevant studies that were not initially found through the database search and that were conducted on this topic. As a consequence of these bibliographic searches, we initially found 5,802 articles. This number includes duplicate hits (e.g., when the same paper was located in both databases). After removing the duplicates, we examined the abstracts of potentially relevant papers to determine whether they met our inclusion criteria. A total of 582 papers remained to be inspected. Based on this set, we eliminated entries that were inconsistent with our eligibility criteria and papers that shared the same dataset of a study already selected for the meta-analysis, such as multiple analyses conducted with an identical dataset on an identical variable pair (*K* = 480). Finally, we contacted authors for additional data in the case of papers that did not include the necessary information to compute the effect sizes. A final set of 102 research articles was included in the current meta-analysis after the application of all the exclusion decisions. The PRISMA diagram in Figure 1 describes how articles were selected and filtered through different phases of the search process, including reasons for excluding articles during the in-depth review stage.

### Coding

From each study, we extracted data regarding: (a) sample size; (b) mean age in the sample; (c) gender (coded as the percentage of women in the sample); (d) type of sample: 1 = student sample, 2 = non-student sample, 3 = representative sample. In addition to this, other more specific coding procedures were applied. With regard to the dependent variable (i.e., intentions, self-reported or actual behavior) we often found articles reporting two or more of these measures. Our strategy was to choose as dependent variable the more “objective” measure included in a given study. For example, if a study included measures of all these three different outcomes (intentions, self-reported behavior, actual behavior), to calculate the effect size we used the actual behavior measure. If we found two of these three outcomes (e.g., intentions and self-reported behavior), we used the self-reported behavior outcome. If the primary study reported both self-reported and actual behavior, we used the actual behavior. In other words, the more “objective” outcome available in each study was been selected for the meta-analysis. Such a strategy allowed us to reduce the number of studies reporting multiple non-independent effect sizes that could affect the final estimates in the current meta-analysis.

### Sensitivity Analysis

Because in some cases the data for the calculation of effect sizes were derived from multivariate analyses (multiple regressions, path models, SEM, etc.), the effect sizes based on r values may be –over- or underestimated. Therefore, we explored, through a sensitivity analysis, if the effect size estimates vary as a function of effect sizes that are zero-order (i.e., derived from univariate analyses) or derived from partial coefficients (see the Statistical tests section for more details). A sensitivity analysis has also been carried out to highlight any eventual difference between studies reporting and not-reporting multiple non-independent effect sizes (see the Results section for more details). Finally, a sensitivity analysis has been carried out to investigate potential differences between studies that use a general measure of awareness of consequences and studies that focus on more specific awareness measures, such as beliefs in climate change (see the Results section for more details).

### Statistical Tests

We used the r correlation coefficient as the effect size metric for the current meta-analysis. For studies that only reported β coefficients we had applied Peterson and Brown (2005) formula: *r* = β + 0.05 λ (where λ = 1 for non-negative βs, and λ = 0 for negative βs) in imputing the corresponding r coefficients. We also computed r values for studies that did not conduct correlational analyses via sample sizes along with *t*-values, χ^2^ values, *p*-values, and standardized mean differences (i.e., Cohen's d). In addition, we reverse-scored several measurements to assure that each positive effect size computed would represent a direct positive association between the various predictors (attitude, intentions, values, awareness, and emotions) and energy-saving behavior (ESB). We adopted a random-effects model to calculate the aggregated effect size of each predictor on ESB. Because our sample contained studies conducted with noticeably different features, we did not used a fixed-effect model. In fact, the latter model assumes that all the studies included are functionally identical and share a single canonical effect size (Hedges and Vevea, 1998; Borenstein et al., 2010). In addition to relaxing this assumption, the random-effects model allows for more unconditional inferences (i.e., a generalizable conclusion to situations beyond the sampled studies) of the results (Field, 2001). Even though it was not very frequent, sometimes we found studies reporting non-independent effect sizes (e.g., multiple measures of the same variable). In these cases, we computed effect sizes using Cooper's (1998) Shifting-Unit-of-Analysis method for studies that report multiple, non-independent effect sizes. As such, we referred to the study as the unit of analysis meaning that each study included would contribute only to one summary effect size to the main analysis (see Cooper, 1998; see also the sensitivity analysis paragraph for more details about this point). We display the 95% confidence intervals alongside indices of heterogeneity assessment like *I*^2^, i.e., the cross-studies “inconsistency index” (Higgins and Thompson, 2002; Higgins et al., 2003), Cochran Q, and tau-squared (the “study-to-study variances”; Borenstein et al., 2009). We also addressed publication bias by examining the funnel plots, where all effect sizes are plotted against the standard error. To check for a potential publication bias, we visually inspected the symmetry of the funnel plots. We also examined the classical Rosenthal's (1979) fail-safe N. We applied the mixed-effects model in the categorical univariate moderator analyses and the meta-regression analyses for the continuous moderators. All analyses in the current meta-analysis were conducted using the Comprehensive Meta-Analysis (CMA) software, Version 3.0 (Borenstein et al., 2009, 2014).

## Results

### Sensitivity Analysis

Results of the sensitivity analysis did not show differences among the sub-groups of effect size estimates derived from univariate analyses (i.e., zero-order) vs. from multivariate analyses (i.e., partial coefficients), across each predictor (all *p*s = ns). Likewise, the sensitivity analysis did not show differences among the sub-groups of studies reporting vs. not-reporting multiple non-independent effect sizes, across each predictor (all *p*s = ns). Finally, results of the sensitivity analysis concerning differences between studies employing measures of general or specific measures of awareness are reported in the next sections (i.e., *Awareness* section). In the following sections, we describe the results on the estimation of average effect size of predictor-criterion relations, publication bias, and relevant moderators.

### Attitudes: Overall and Publication Bias Results

The estimated effect sizes of the association between attitudes and energy-saving behaviors (or intentions) are displayed in Table 1.

**Table 1:** Summary of ES of the association between attitudes and energy saving behaviors (or intentions).

	**Statistics for each study**			
**References**	**Sample** **size**	**Correlation**	**95%** **LLCI**	**95%** **ULCI**
Afroz et al. (2015a) (ESPR Journal)	350	0.20	0.10	0.30
Aini et al. (2013)	201	0.14	0.00	0.27
Al-Amin et al. (2016)	300	0.30	0.19	0.40
Barbarossa et al. (2015)	611	0.67	0.62	0.71
Barbarossa et al. (2015)	600	0.77	0.74	0.80
Barbarossa et al. (2015)	794	0.73	0.70	0.76
Carmi et al. (2015)	1,160	0.26	0.21	0.31
Claudy et al. (2013)	254	0.34	0.23	0.44
Craig and Allen (2014)	2,058	0.78	0.77	0.80
Degirmenci and Breitner (2017)	167	0.33	0.19	0.46
Dixon et al. (2015)	2,919	0.14	0.10	0.17
Engelken et al. (2016)	109	0.74	0.64	0.81
Fornara et al. (2016)	432	0.31	0.22	0.39
Gaspar and Antunes (2011)	1,303	0.19	0.13	0.24
Halder et al. (2016)	402	0.64	0.58	0.70
Halder et al. (2016)	130	0.55	0.42	0.66
Han et al. (2017)	607	0.77	0.74	0.80
Hansla et al. (2008)	855	0.42	0.36	0.47
Hatzl et al. (2014)	58	0.21	−0.05	0.44
Hertel and Menrad (2016)	104	0.51	0.35	0.64
Kim et al. (2014)	1,647	0.61	0.58	0.64
Klöckner et al. (2013)	1,787	0.22	0.17	0.26
Korcaj et al. (2015)	200	0.40	0.28	0.51
Lin and Syrgabayeva (2016)	305	0.32	0.22	0.42
Litvine and Wüstenhagen (2011)	170	0.26	0.11	0.40
Mohamed et al. (2016)	3,505	0.72	0.71	0.74
Moons and De Pelsmacker (2012)	1,199	0.56	0.52	0.60
Murtagh et al. (2013)	83	0.46	0.27	0.61
Nayum and Klöckner (2014)	1,517	0.18	0.13	0.23
Nguyen et al. (2016)	682	0.29	0.22	0.36
Park and Ohm (2014)	1,429	0.50	0.46	0.54
Pettifor et al. (2015)	295	0.11	0.00	0.23
Prete et al. (2017)	128	0.58	0.45	0.68
Rai and Beck (2017)	522	0.38	0.30	0.45
Scott et al. (2014)	279	0.87	0.84	0.90
Shi et al. (2017)	580	0.70	0.66	0.74
Wittenberg and Matthies (2016)	213	0.48	0.37	0.58
Wolske et al. (2017)	904	0.44	0.39	0.49
Yang et al. (2016)	526	0.30	0.22	0.37
Yun and Lee (2015)	753	0.77	0.73	0.79
Zierler et al. (2017)	628	0.15	0.07	0.22

*A 95% CI that does not include zero provides evidence of a significant effect*.

The analysis revealed a moderate/large positive association between attitude and ESB: *r* = 0.482; 95% CI (confidence interval) lower limit (LLCI)/upper limit (ULCI) = 0.396/0.559; *p* < 0.001. We observed a non-negligible level of variation in the distribution of effect sizes (Tau = 0.343, Tau-squared = 0.117). This might be explained by the considerable extent of heterogeneity [i.e., *I*^2^ = 98.84; Q(40) = 3458.58, *p* = 0.0001] inherent among the sampled studies.

**Figure 2:**
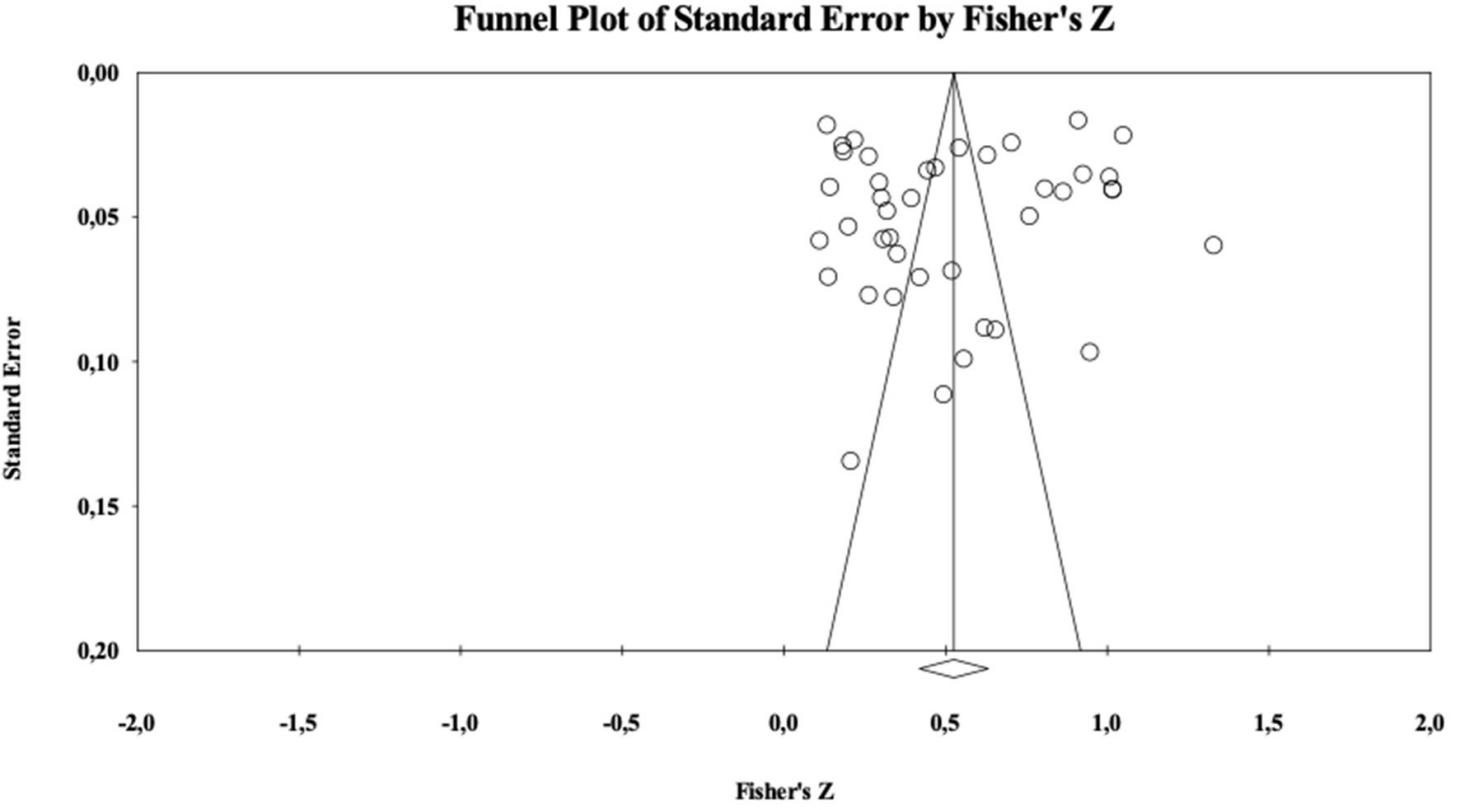
Funnel plot for attitudes.

To address the extent of publication bias we first examined the classical Rosenthal's (1979) fail-safe N. This index estimates how many unpublished studies with a null effect size would be necessary to turn a significant population effect size estimate into a non-significant one based on the Stouffer Z-test. Rosenthal (1979) recommended the fail-safe N to be smaller than a 5K+10 benchmark. In our meta-analysis, for the relationship between attitudes and ESB, the critical value 5K+10 was 215. The analyses showed a Nfs = 75,246. Moreover, we inspected the so-called “funnel plot,” that is a graphical technique in which the standard error of each study's effect size is plotted against the standardized effect size itself. Lack of publication bias is suggested by a symmetrical cloud of studies centered around the population effect size, with increasing variability at increasing levels of standard error. This is because there should be about as many studies providing non-significant results as those providing significant ones at each specific level of standard error, whereas studies with smaller standard errors should also be closer to the population effect size. As shown in the Figure 2, the funnel plot has a rather symmetrical shape. In sum, both these indicators suggest that the present analysis is not contaminated by publication bias.

### Attitudes: Moderation Effects

For all the predictors, we used the percentage of women in the sample as a continuous variable to be included in a meta-regression model that aims to estimate the potential moderating effect of gender on the relationship between the independent variable and ESB. In the case of attitudes, results show no significant moderating effect of gender (β = 0.001, *p* = ns). A similar meta-regression model was conducted considering participants' age as moderator in the relationship between ecological attitude and ESB. Results show no significant moderating effect of age on the relationship between ecological attitude and ESB (β = 0.001, *p* = ns). Concerning the different types of sample (i.e., students vs. non-students vs. representative sample; see the previous section), results did not show a significant moderating role of this factor, Q(1) = 0.014, *p* = ns.

Interestingly, results showed a significant moderating role of the type of dependent variable considered in the study. Associations with attitudes were significant for studies that considered intentions (*r* = 0.565, LLCI/ULCI = 0.475/0.643) and self-reported behavior as outcomes (*r* = 0.312, LLCI/ULCI = 0.147/0.460). On the contrary, the association with attitudes was not significant in the case of studies that considered actual behavior as outcome (*r* = 0.338, LLCI/ULCI = −0.099/0.666), Q(2) = 9.03, *p* < 0.01. Moreover, results showed that the effect size of the association between attitudes and intention (*r* = 0.565, LLCI/ULCI = 0.475/0.643) is significantly larger than the effect size of the association between attitudes and self-reported behavior (*r* = 0.312, LLCI/ULCI = 0.147/0.460), Q(1) = 8.40, *p* < 0.01.

### Intentions: Overall and Publication Bias Results

To assess the strength of the association between intentions to adopt energy-saving solutions and energy-saving behaviors, we considered in the current meta-analysis only those studies that measured actual or self-reported ESBs as outcomes. The estimated effect sizes are displayed in Table 2.

**Table 2:** Summary of ES of the association between intentions to adopt energy saving solutions and energy saving behavior.

	**Statistics for each study**			
**References**	**Sample** **size**	**Correlation**	**95%** **LLCI**	**95%** **ULCI**
Afroz et al. (2015a) (ESPR Journal)	350	0.32	0.22	0.41
Ajzen et al. (2011)	79	0.62	0.46	0.74
Akman and Mishra (2015)	157	0.25	0.10	0.39
Al-Amin et al. (2016)	300	0.28	0.17	0.38
Azar and Al Ansari (2017)	227	0.56	0.46	0.64
Carmi et al. (2015)	1,160	0.18	0.12	0.24
Dixon et al. (2015)	2,919	0.24	0.21	0.27
Gerpott and Paukert (2013)	453	0.23	0.14	0.32
Hatzl et al. (2014)	58	0.31	0.05	0.52
Khorasanizadeh et al. (2016)	221	0.44	0.33	0.54
Klöckner et al. (2013)	1,787	0.33	0.28	0.37
Murtagh et al. (2013)	83	0.15	−0.07	0.35
Nayum and Klöckner (2014)	1,517	0.34	0.30	0.39
Rai and Beck (2017)	522	0.11	0.02	0.19
Webb et al. (2013)	200	0.25	0.12	0.38
Zierler et al. (2017)	628	0.27	0.19	0.34

*A 95% CI that does not include zero provides evidence of a significant effect*.

The analysis revealed a moderate positive association between intention and ESB: *r* = 0.300; 95% CI LLCI/ULCI = 0.249/0.350; *p* < 0.0001. We observed a non-negligible level of variation in the distribution of effect sizes (Tau = 0.096, Tau-squared = 0.009). This might be explained by the moderate/large extent of heterogeneity [i.e., *I*^2^ = 84.65; Q(15) = 97.76, *p* = 0.0001] among the sampled studies.

**Figure 3:**
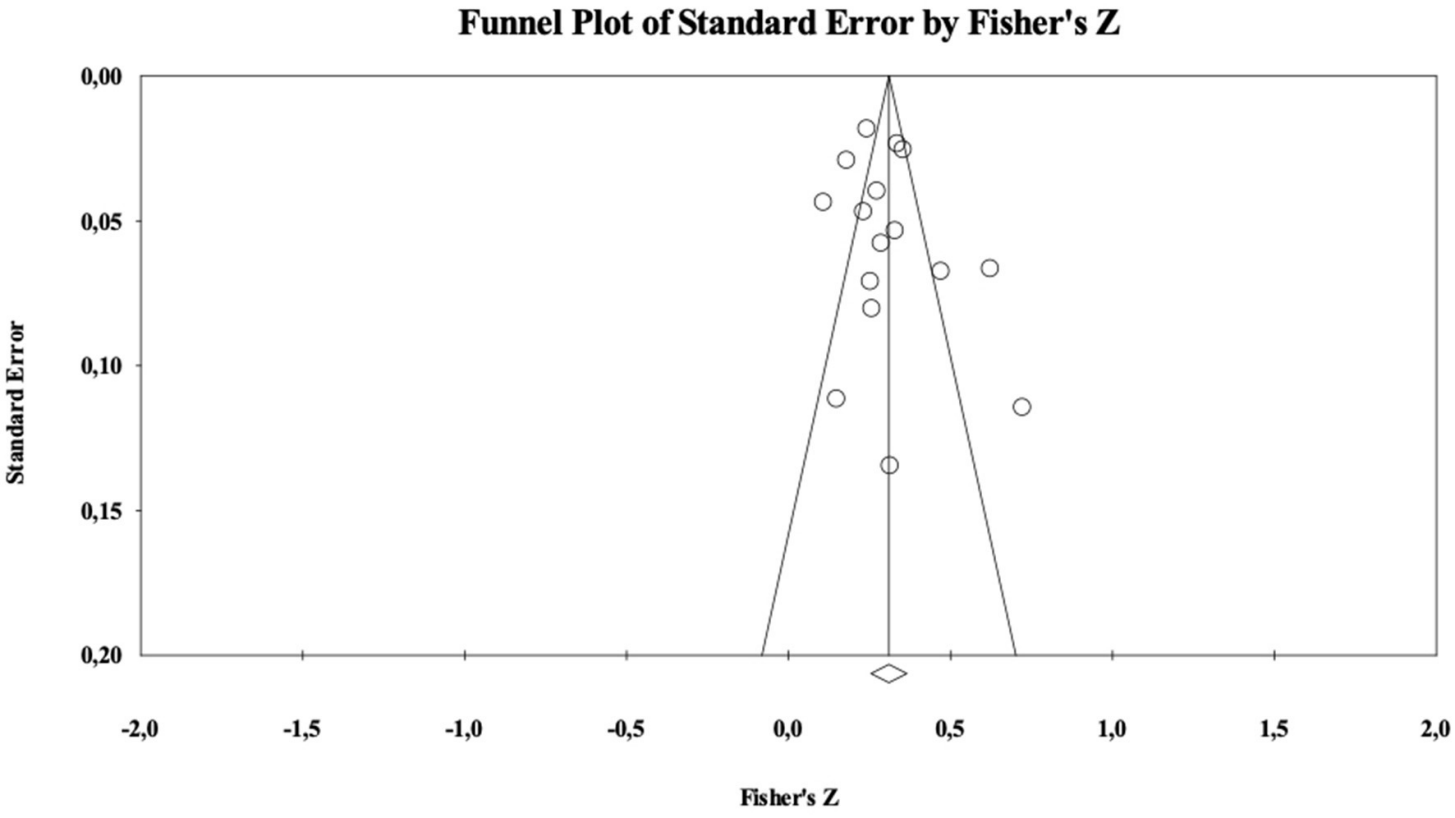
Funnel plot for intentions.

The critical value 5K+10 of Nfs was 90. The analyses showed a Nfs = 2,925. As showed in Figure 3, the funnel plot is rather symmetrical. In sum, both these indicators suggest that the present analysis is not contaminated by publication bias.

### Intentions: Moderation Effects

Results showed no significant moderating effects of gender (β = 0.001, *p* = ns) and age (β = −0.003, p = ns) on the relationship between intentions and ESB. A significant moderating effect of sample type emerged (students vs. non-students): Q(1) = 4.55, *p* < 0.01. Although associations were significant for both student (*r* = 0.421, LLCI/ULCI = 0.300/0.529) and non-student samples (*r* = 0.274, LLCI/ULCI = 0.214/0.333), the effect size was significantly larger in the former case. Regarding the type of dependent variable (actual vs. self-reported behavior), no significant moderation effects were shown [Q(1) = 0.61, *p* = ns].

### Values: Overall and Publication Bias Results

The estimated effect sizes of the association between values and energy saving behaviors (or intentions) are displayed in Table 3.

**Table 3:** Summary of ES of the association between values and energy saving behaviors (or intentions).

	**Statistics for each study**			
**References**	**Sample** **size**	**Correlation**	**95%** **LLCI**	**95%** **LCI**
Barbarossa et al. (2017)	2,005	0.36	0.32	0.40
Fornara et al. (2016)	432	0.06	−0.03	0.15
Girod et al. (2017)	1,101	0.37	0.32	0.42
Hatzl et al. (2014)	56	0.22	−0.04	0.46
Murtagh et al. (2013)	83	0.14	−0.08	0.35
Nayum et al. (2016)	1,508	0.27	0.23	0.32
Yang et al. (2016)	526	0.33	0.25	0.40

*A 95% CI that does not include zero provides evidence of a significant effect*.

The analysis revealed a small/moderate positive association between pro-environmental values and ESB: *r* = 0.271; 95% CI LLCI/ULCI = 0.193/0.346; *p* < 0.0001. We observed a non-negligible level of variation in the distribution of effect sizes (Tau = 0.097, Tau-squared = 0.009). This might be explained by the moderate/large extent of heterogeneity [i.e., *I*^2^ = 86.93; Q(6) = 45.93, *p* = 0.0001] emerging among the sampled studies.

**Figure 4:**
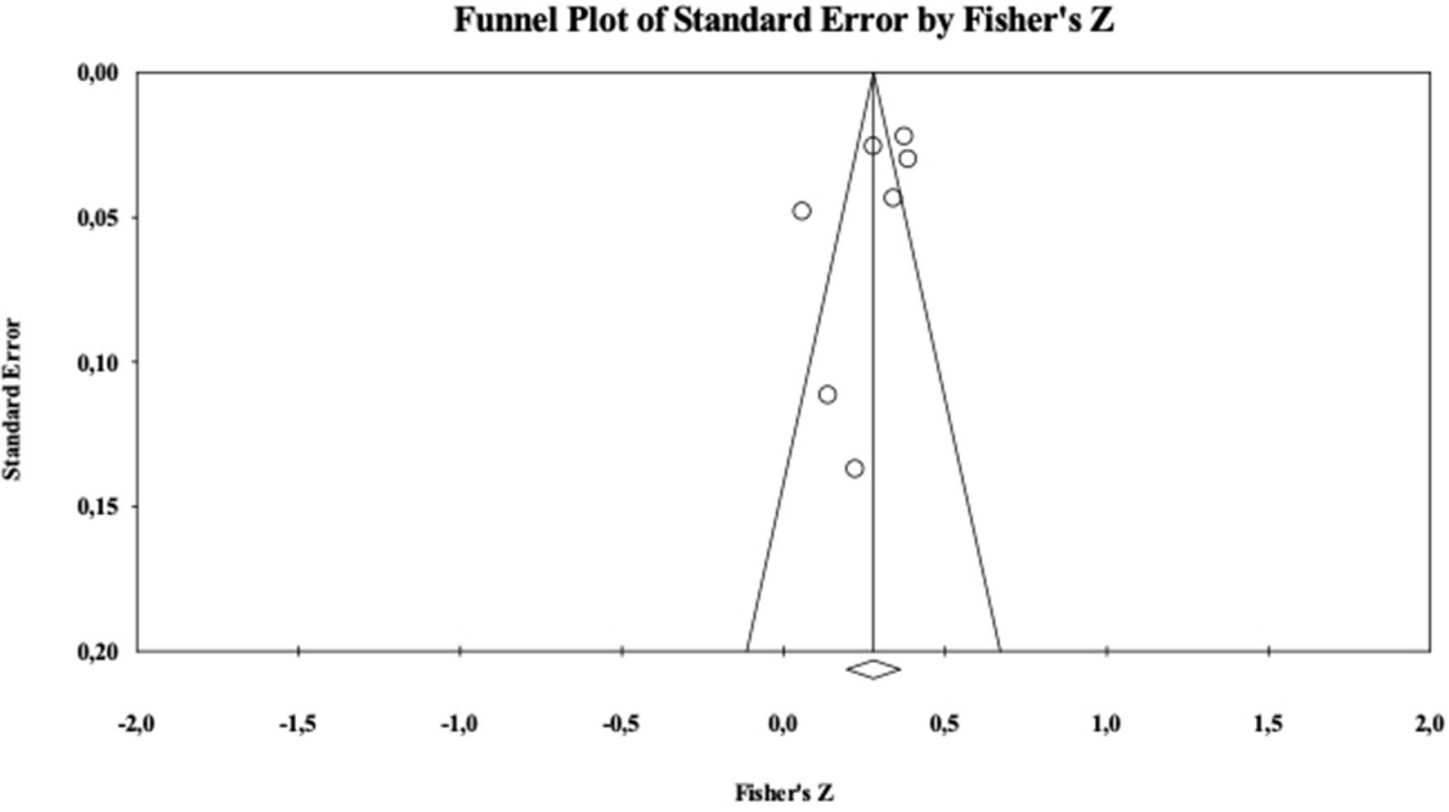
Funnel plot for values.

The critical value 5K+10 of Nfs was 45. Analyses showed a Nfs = 715. As showed in Figure 4 the funnel plot was rather symmetrical. In sum, both these indicators suggest that the present analysis is not contaminated by publication bias.

### Values: Moderation Effects

**Figure 5:**
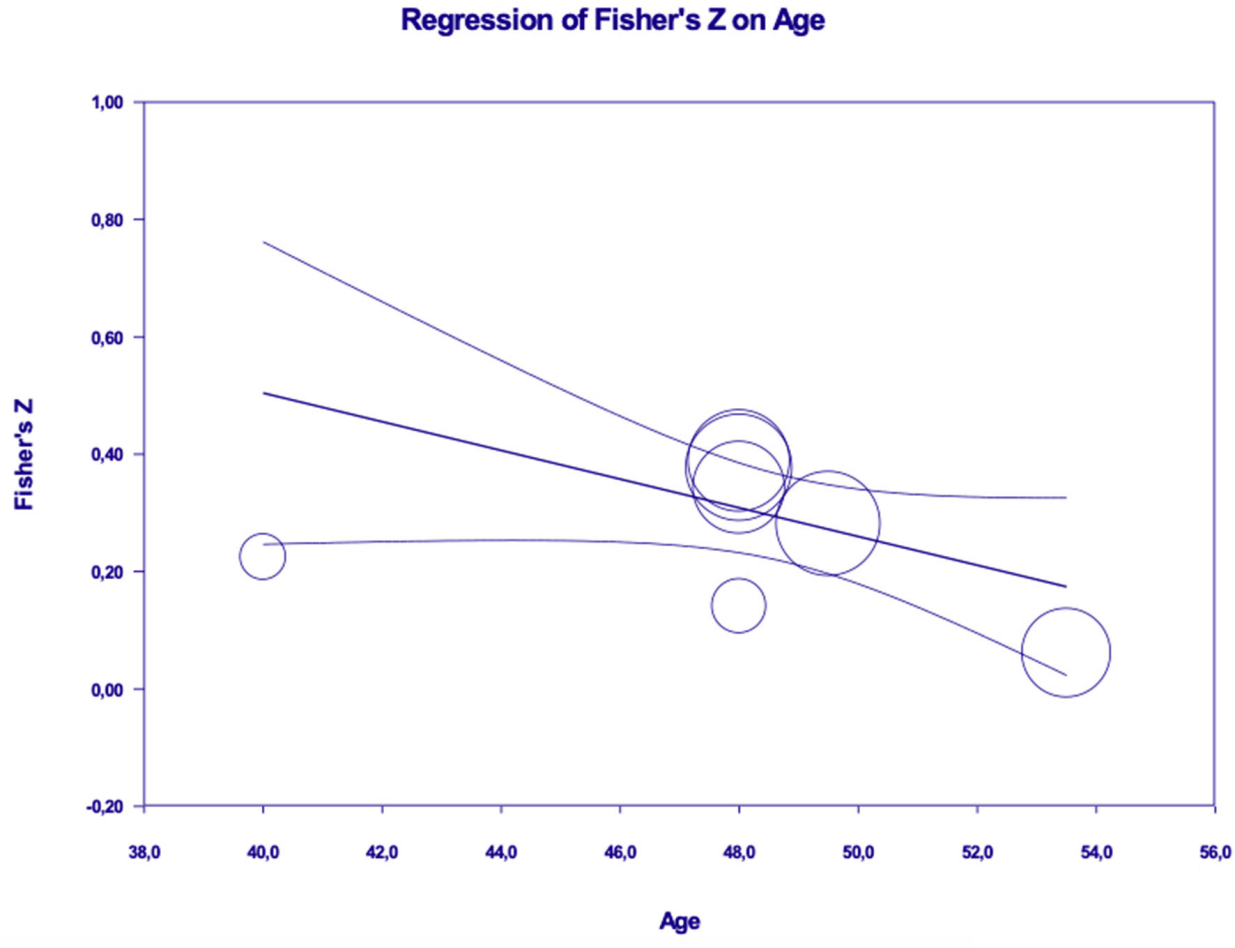
Moderation effect of age in the relation between values and energy saving behaviors (or intentions).

Results revealed a significant moderation effect of age (β = −0.02, *p* < 0.05; *R*^2^ analog = 0.59), with the effect approaching to zero as participants' age increases (See Figure 5). No significant moderation effects emerged for gender (β = −0.002, *p* = ns), type of the sample [Q(1) = 1.25, *p* = ns] and type of dependent variable [Q(2) = 0.79, *p* = ns].

### Awareness: Sensitivity Analysis

As stated before, under the label “Awareness,” we included both studies that dealt with the more general concept of “awareness of consequences of one's own behavior” and studies that dealt with the more specific dimension of “beliefs in climate change.” Therefore, prior to the main effects and moderation tests, we performed a sensitivity analysis to explore whether the effect size in the index of association that was derived from a measure of awareness of consequences (*r* = 0.333, LLCI/ULCI = 0.255/0.407) is different from the effect size derived from a measure of beliefs in climate change (*r* = 0.223, LLCI/ULCI = 0.057/0.378). While both effects were significant, they were not significantly different from each other, Q(1) = 1.512, *p* = 0.219. Thus, we can conclude that the overall effect size of the relationship between this predictor and ESB is not affected from specific measurement features used to assess either awareness of consequences or beliefs in climate change.

### Awareness: Overall and Publication Bias Results

The estimated effect sizes of the association between awareness of consequences/beliefs in climate change and energy saving behaviors (or intentions) are displayed in Table 4.

**Table 4:** Summary of ES of the association between awareness of consequences/beliefs in climate change and energy saving behaviors (or intentions).

	**Statistics for each study**			
**References**	**Sample** **size**	**Correlation**	**95%** **LLCI**	**95%** **ULCI**
Afroz et al. (2015b) (Euasia Journal)	200	0.06	−0.08	0.19
Alam et al. (2014)	200	0.41	0.28	0.52
Barbarossa et al. (2015)	611	0.48	0.42	0.54
Barbarossa et al. (2015)	600	0.48	0.42	0.54
Barbarossa et al. (2015)	794	0.31	0.25	0.37
Barbarossa et al. (2017)	2,005	0.50	0.47	0.53
Bichard and Kazmierczak (2012)	671	0.19	0.11	0.26
Engelken et al. (2016)	109	0.31	0.13	0.47
Fornara et al. (2016)	432	0.26	0.17	0.34
Gerpott and Paukert (2013)	453	0.40	0.32	0.47
Hansla et al. (2008)	855	0.24	0.17	0.30
He and Zhan (2017)	396	0.49	0.41	0.56
Hobman and Frederiks (2014)	1,154	0.08	0.02	0.13
Karytsas and Theodoropoulou (2014)	201	0.03	−0.11	0.17
Klöckner et al. (2013)	1,787	0.22	0.18	0.26
Lillemo (2014)	1,004	0.42	0.37	0.47
Lin and Syrgabayeva (2016)	305	0.23	0.12	0.33
Menon and Mahanty (2016)	1,017	0.55	0.51	0.59
Nakada et al. (2016)	4,750	0.10	0.07	0.13
Nayum and Klöckner (2014)	1,517	0.25	0.20	0.30
Sapci and Considine (2014)	602	0.34	0.27	0.41
Spence et al. (2010)	1,491	0.20	0.15	0.25
Tsagarakis et al. (2011)	1,440	0.15	0.10	0.20
Vaccaro and Echeverri (2010)	1,257	0.71	0.68	0.74
Wang et al. (2011)	816	0.24	0.17	0.30
Wang et al. (2017)	253	0.51	0.41	0.60
Wolske et al. (2017)	904	0.24	0.18	0.30
Li et al. (2013)	1,516	0.34	0.29	0.38
Zhang X. et al. (2013)	349	0.13	0.03	0.23
Zhang Y. et al. (2013)	273	0.13	0.01	0.24

*A 95% CI that does not include zero provides evidence of a significant effect*.

Results revealed a moderate positive association between awareness and ESB: *r* = 0.311; 95% CI LLCI/ULCI = 0.241/0.379; *p* < 0.001. We observed a non-negligible level of variation in the distribution of effect sizes (Tau = 0.209, Tau-squared = 0.044). This might be explained by the considerable extent of heterogeneity [i.e., *I*^2^ = 97.51; Q(29) = 1168.14, *p* = 0.0001] across the sampled studies.

**Figure 6:**
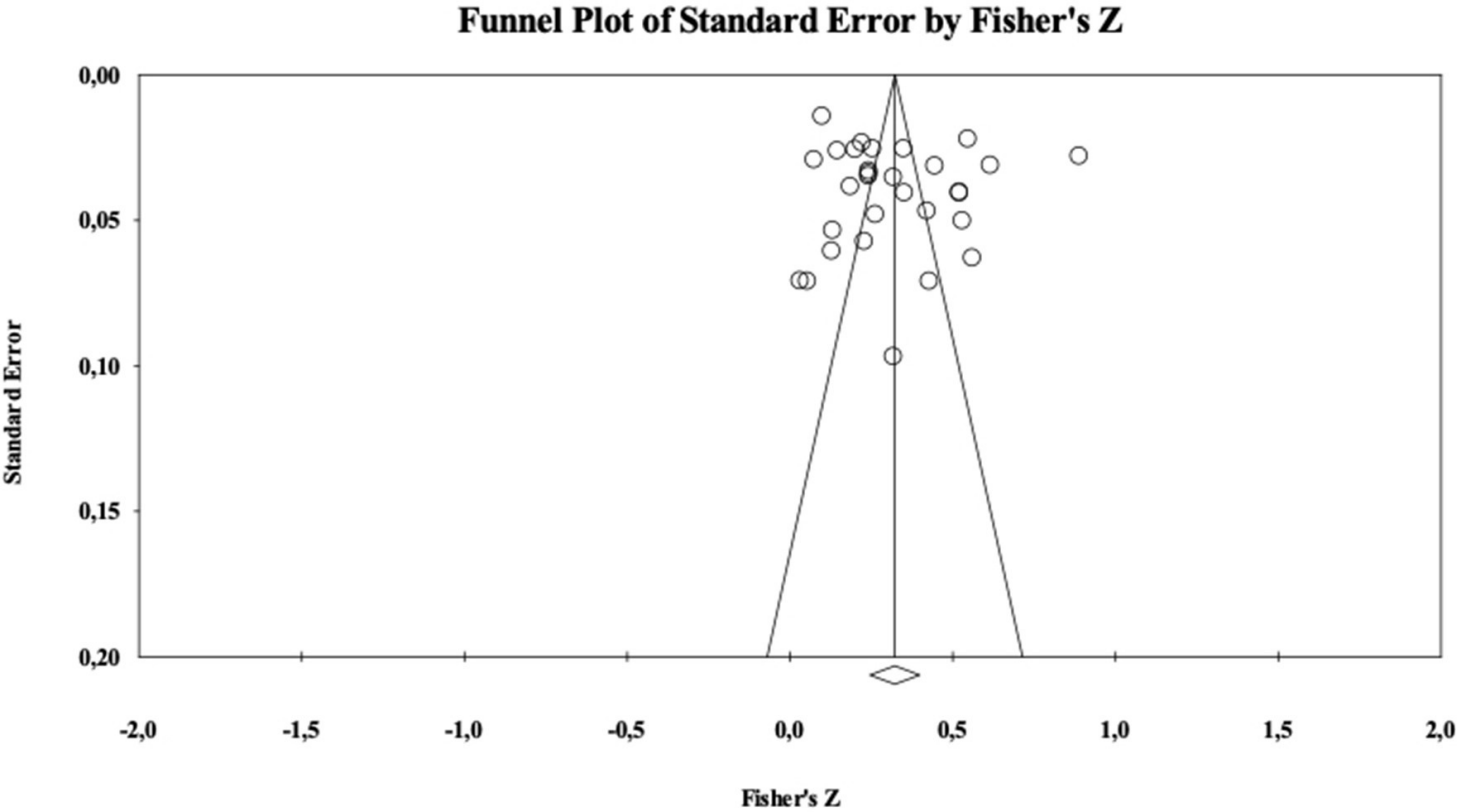
Funnel plot for awareness.

The critical values 5K+10 of Nfs was 160. Analyses showed such a Nfs = 8,803. As showed in the Figure 6, the funnel plot reveals a rather symmetrical distribution. In sum, both these indicators suggest that the present analysis is not likely to be contaminated by publication bias.

### Awareness: Moderation Effects

Results revealed no significant moderating effects in the relation between awareness and ESB for gender (β = −0.001, *p* = ns), age (β = 0.001, *p* = ns), sample typology [Q(1) = 0.70, *p* = ns] and type of dependent variable [Q(2) = 0.08, *p* = ns].

### Emotions: Overall and Publication Bias Results

The estimated effect sizes of the association between emotions and energy saving behaviors (or intentions) are displayed in Table 5.

**Table 5:** Summary of ES of the association between emotions and energy saving behaviors (or intentions).

	**Statistics for each study**			
**References**	**Sample** **size**	**Correlation**	**95%** **LLCI**	**95%** **ULCI**
Fornara et al. (2016)	432	0.32	0.23	0.40
Han et al. (2017)	607	0.66	0.61	0.70
Moons and De Pelsmacker (2012)	1,199	0.60	0.56	0.63
Taufik et al. (2016)	152	0.61	0.50	0.70
Taufik et al. (2016)	132	0.85	0.79	0.89
Wang and Wu (2016)	775	0.27	0.20	0.33
Webb et al. (2013)	200	0.46	0.34	0.56
Wolske et al. (2017)	904	0.25	0.19	0.31

*A 95% CI that does not include zero provides evidence of a significant effect*.

As the number of studies on single discrete emotions (e.g., pride, guilt, or anger) was rather limited, in our meta-analysis we pooled all these emotions together as potential predictors of ESB. This was possible because, independently from the emotional valence, each study included here considered these emotions as drivers of ESB. Results revealed a large positive association between emotions (e.g., guilt, pride, etc.) and ESB, r = 0.533, 95% CI LLCI/ULCI = 0.379/0.658, *p* = 0.0001. We observed a non-negligible level of variation in the distribution of effect sizes (Tau = 0.276, Tau-squared = 0.076). This might be explained by the considerable extent of heterogeneity [i.e., *I*^2^ = 97.49; Q(7) = 279.62, *p* < 0.0001] inherent among the sampled studies.

**Figure 7:**
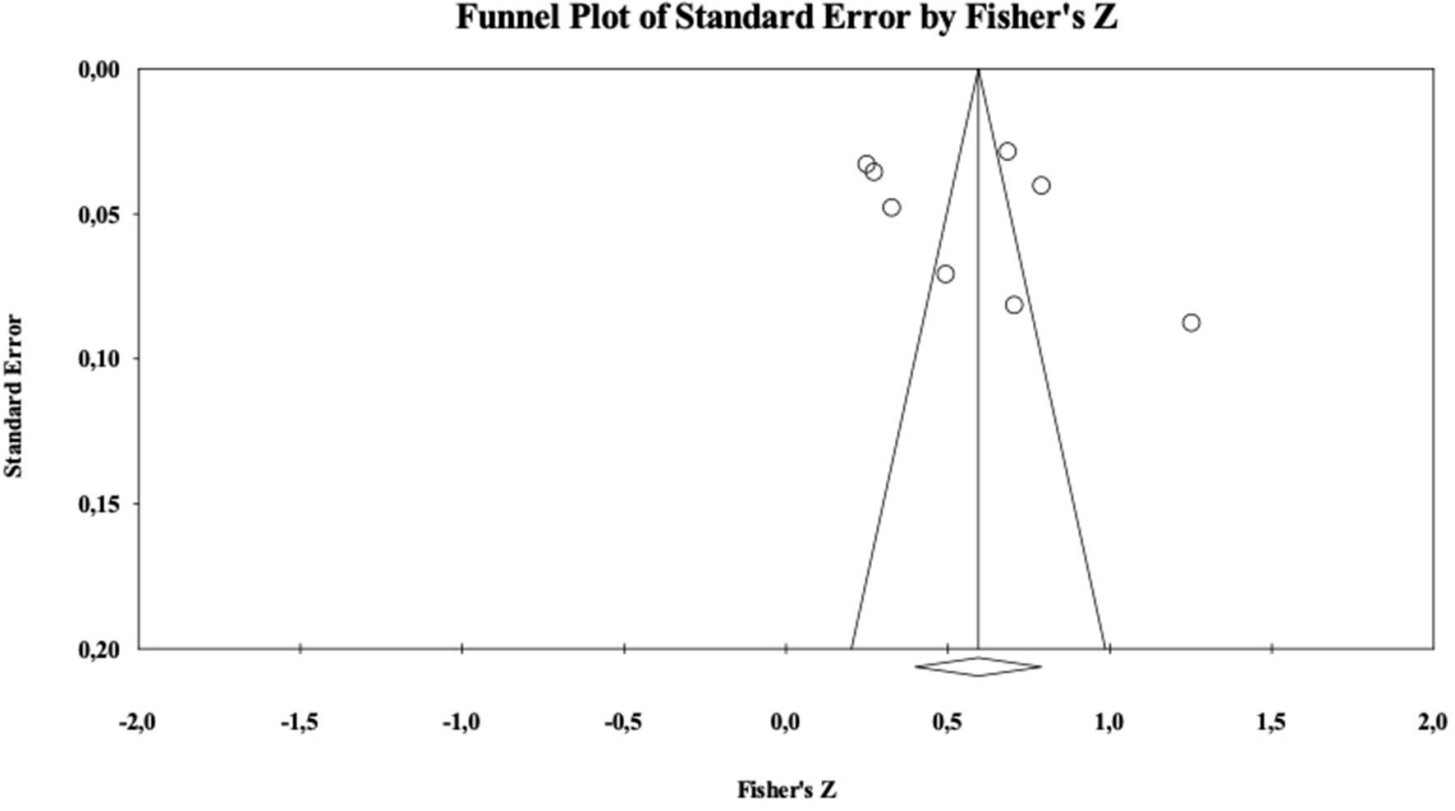
Funnel plot for emotions.

The critical values 5K+10 of Nfs was 50. Analyses showed a Nfs = 2,357. As showed in Figure 7, the funnel plot was rather symmetrical. In sum, both these indicators suggest that the present analysis is not contaminated by publication bias.

### Emotions: Moderation Effects

**Figure 8:**
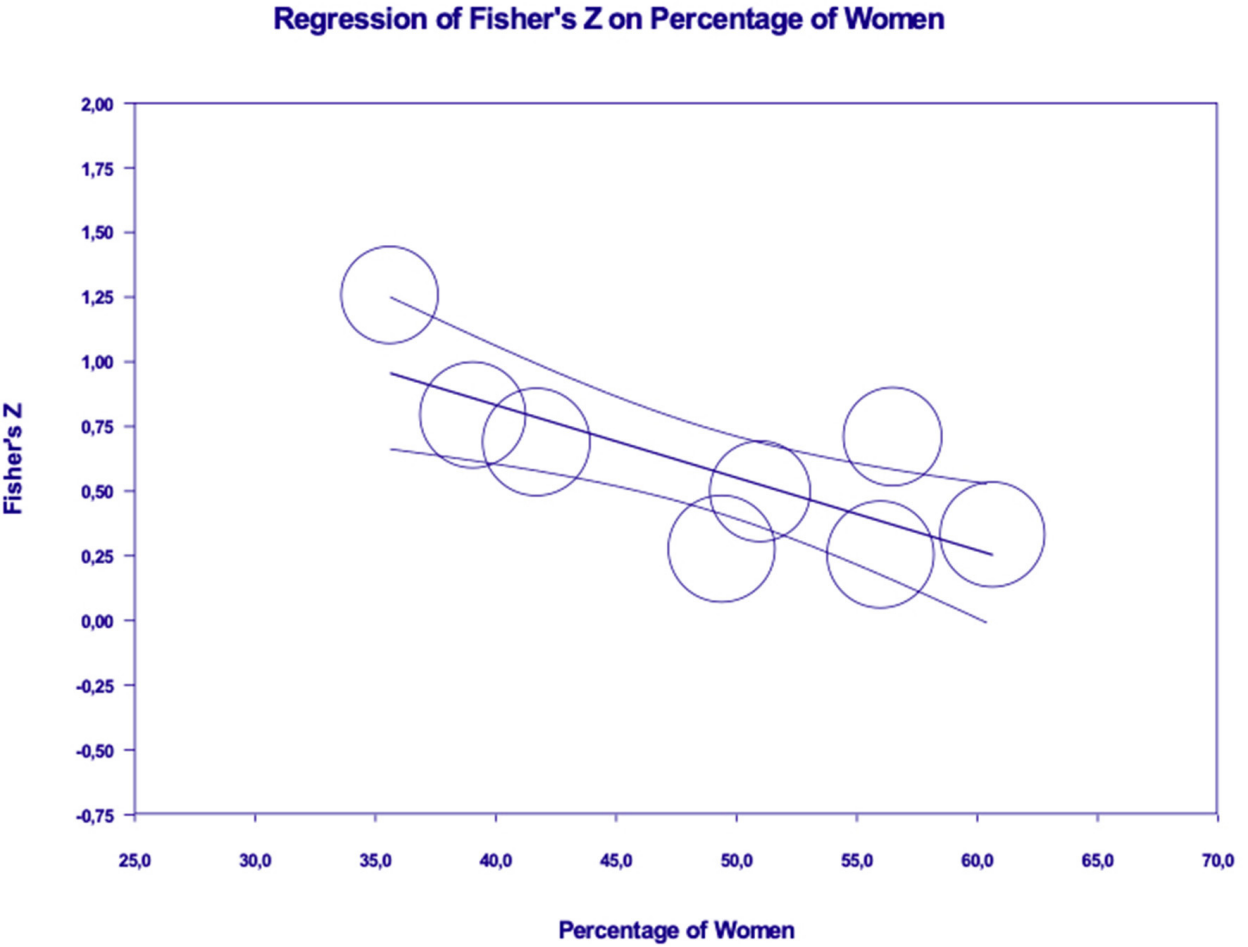
Moderation effect of gender in the relation between emotions and energy saving behaviors (or intentions).

**Figure 9:**
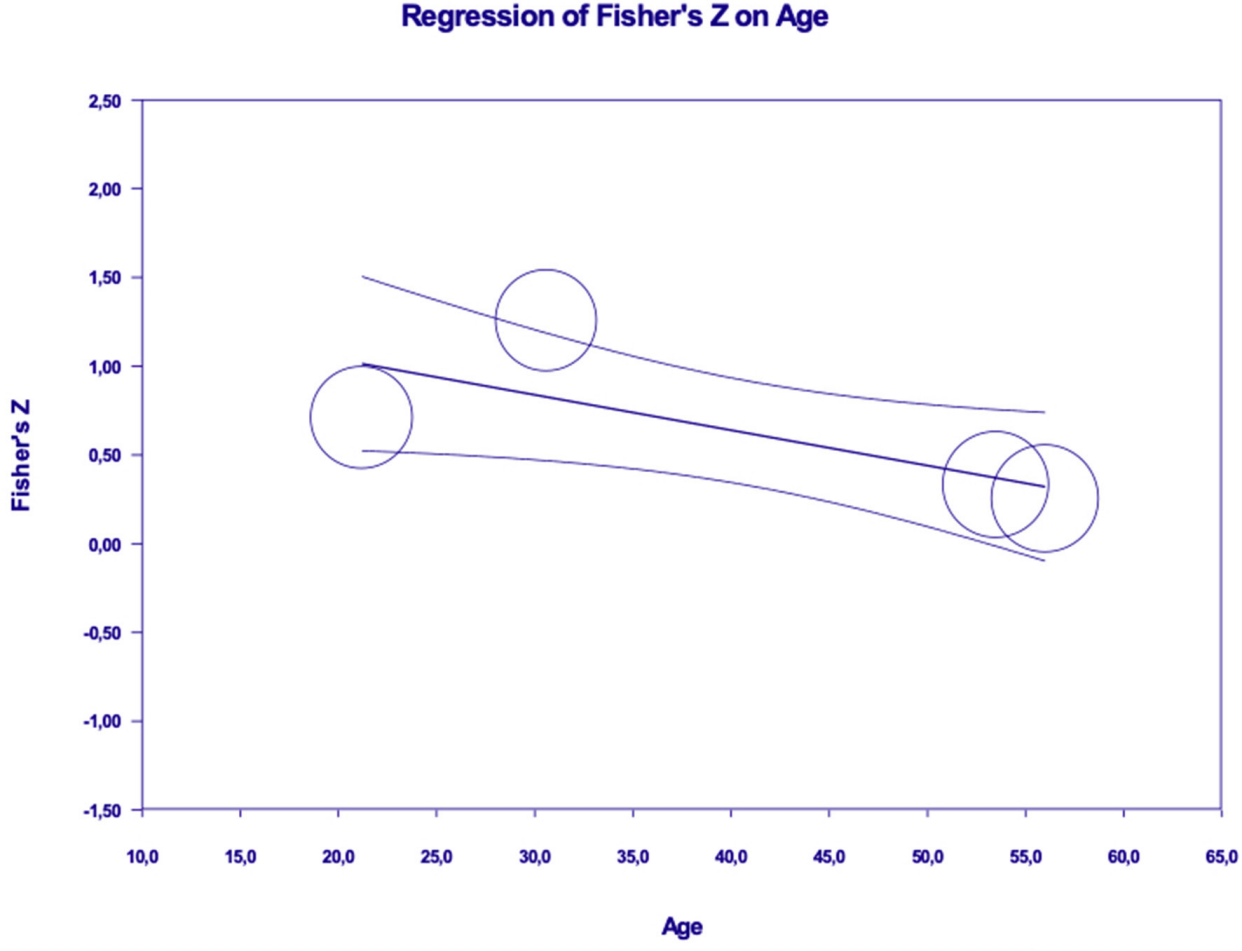
Moderation effect of age in the relation between emotions and energy saving behaviors (or intentions).

Results revealed a significant moderation effect, in the relation between emotions and ESB, for gender (β = −0.03, *p* < 0.001; *R*^2^ analog = 0.60) and age (β = −0.02, *p* < 0.05; *R*^2^ analog = 0.59), with the effects approaching to zero as the percentage of women and participants' age increase (see Figures 8, 9). Results did not show a significant moderation effect for sample type [Q(1) = 0.176, *p* = ns] and type of dependent variable [Q(1) = 0.124, *p* = ns].

## Discussion, Conclusions, and Practical Implications

Taken together, results indicate that the five classes of psychological factors considered in this meta-analysis are positive and significant predictors of energy saving behaviors (and intentions).

We show a large association of energy saving behaviors with positive and negative emotions (such as guilt, anger or pride); a positive moderate/large association of energy saving behaviors with pro-environmental attitudes; a positive moderate association of energy saving behaviors with awareness of consequences/beliefs in climate change; a positive moderate association of energy saving behaviors with intentions to adopt energy saving solutions; a positive small/moderate association of energy saving behaviors with pro-environmental values. Thus, while all the potential determinants included in our study might be important to explain energy saving behaviors, some predictors, like emotions, show more explanatory power than others, like values or beliefs. It is difficult to explain these differences, without a direct empirical comparison of the mechanisms involved in such relations. On a speculative level, one might argue that pro-environmental beliefs or biospheric values are widely shared in contemporary society, at a global level (particularly among respondents that usually participate in psychological studies); thus, it might be hard to explain differences in human actions on that basis. Also, attitude-behavior or value-behavior gaps are not novel in social psychological or sociological research. Conversely, affective states or emotions associated to a particular course of action in the energy domain (or in the environmental domain in general), might be more directly associated to real-life choices, especially when individuals are asked to change habitual or routinary patterns of behaviors (see also Carrus et al., 2020).

Our moderation analyses also uncovered some interesting results. Participants' age emerged as a relevant moderator in the associations of pro-environmental values and emotions with energy saving behaviors (or intentions) suggesting that the role of these variables is weaker among older people.

In the case of emotions, gender also emerged as a significant moderator, suggesting that associations between emotions (such as guilt or pride) and energy-related behaviors are weaker among women, compared to men.

Both the tests of the direct effect sizes and the moderation analyses might have interesting practical implications. In particular, regarding the moderation effects of age in the case of values and emotions, our results suggest that these variables could represent key target factors for intervention strategies addressed to younger generations. Likewise, the moderation effect of gender in the association between energy saving behavior and emotions, suggest how these might be a specific factor to be addressed in practical interventions or persuasion campaigns designed purposively to influence energy choices among men, rather than women. Once again, it is not easy to provide a clearcut explanation for these moderation effects, particularly in the case of gender differences: certainly, understanding age and gender differences in the determinants of energy-related choices is an interesting issue for future investigation.

Moderation effects by the typology of the sample recruited (e.g., student vs. non-student) and type of outcome measure (actual vs. self-reported behavior vs. behavioral intentions) are also interesting to discuss. Moderation effects by sample type showed larger effect sizes in student samples compared to non-student samples. Moderation effects by the type of outcome measure (actual vs. self-reported behavior vs. behavioral intentions) when assessing the attitude-behavior links suggest that attitudes are a significant predictor of both intentions and (to a lesser extent) of self-reported behavior. Converesely, our analysis suggests that attitudes might not be a good predictor of actual energy use (e.g., actual electricity consumption measured in kWh).

These kind of moderation effects suggest the existence of both conceptual and methodological issues in current social psychological research on energy saving behaviors (and in general). While it is out of the scope of this paper to discuss the reliability of self-reports in psychological investigation, or the fundamental aspects of the intention-behavior links, it is certainly possible to take these results as an interesting input for the debate on the ecological validity of psychological studies in general, and as a contribution to the necessity to embrace a deeper and more open self-reflexive stance on the quality of research practices in environmental, social and cognitive psychology.

Some limitations of the present study must also be acknowledged: for example, our meta-analytical tests of the effect sizes for values and emotions are based on a relatively limited number of studies. This suggest that these factors could have been under-investigated, at least in the temporal range that we considered here, and in published studies: it might be the case that considering more recent studies and/or including “gray” literature in future meta-analysis could complement the present findings. This fact seems quite surprising in the case of values (a wide investigated variable in environmental psychological research), but less so in the case of emotions, which, on the contrary, have been rather neglected by people-environment studies in the past (e.g., Damasio, 1994; Carrus et al., 2008). This aspect suggests the need for more environmental psychological research on emotions and energy use, especially because emotions emerged from our meta-analysis as the factor having the largest effect size in relation to energy saving. Emotions are an essential motivational driver of human behavior and should thus be considered as a relevant tool to leverage people's transition to more sustainable energy-related decisions.

Another limitation is represented by our choice of the specific predictors to be included in the meta-analysis. Our choice was based on a previous exploration of the literature on energy choices and pro-environmental behaviors, as well as on widely known models of human deliberate action in the environmental domain (e.g., the Theory of Planned Behavior or the Value-Belief-Norm theory). However, other important variables could have been included in our analysis, such as for example personality traits, motives, skills, risk perception, or perception of costs and benefits: future meta-analysis or systematic reviews are thus needed to assess also the role of these factors in energy-related decisions.

In sum, we can conclude that, taken together results of the meta-analyses presented in this paper could have relevant applied implications for both academics and policy makers, as they can provide relevant insights to improve future studies on the psychological determinants of energy saving behaviors, and provide guidelines to tailor specific policies, intervention programs and public campaigns for changing human energy-related behaviors and promoting a sustainable energy transition.

## Data Availability Statement

The data analyzed in this study is subject to the following licenses/restrictions: MA data available on request to the corresponding author. Requests to access these datasets should be directed to giuseppe.carrus@uniroma3.it.

## Author Contributions

GC supervised the conception of the meta-analysis, conduction of the study, and contributed to the writing and revision of the manuscript. LT contributed to the data acquisition, coding, writing, and revision of the manuscript. AP performed the statistical tests and contributed to the conception of the meta-analysis, conduction of the study, writing, and revision of the manuscript. PC, IF, CK, SM, TM, and SV contributed to the writing and revision of the manuscript. All authors contributed to the article and approved the submitted version.

## Conflict of Interest

The authors declare that the research was conducted in the absence of any commercial or financial relationships that could be construed as a potential conflict of interest.
